# Frequent functional activation of RAS signalling not explained by RAS/RAF mutations in relapsed/refractory multiple myeloma

**DOI:** 10.1038/s41598-018-31820-9

**Published:** 2018-09-10

**Authors:** Kwan Yeung Wong, Qiumei Yao, Ling-Qing Yuan, Zhenhai Li, Edmond Shiu Kwan Ma, Chor Sang Chim

**Affiliations:** 1 Department of Medicine, Queen Mary Hospital, The University of Hong Kong, Pokfulam Road, Pokfulam, Hong Kong; 2 Department of Metabolism and Endocrinology, The Second Xiangya Hospital, Central South University, Changsha, Hunan China; 3 Department of Pathology, Hong Kong Sanatorium & Hospital, Happy Valley, Hong Kong

## Abstract

*RAS* mutations are frequent in relapsed/refractory multiple myeloma (RRMM) but functional study in primary samples is scanty. Herein, in primary myeloma plasma cells of 17 suspected RRMM, functional activation of RAS signalling was studied by Western blot of phosphorylated ERK1/2 (phospho-ERK1/2). Moreover, activating mutations in *KRAS*, *NRAS*, *BRAF*, and *ALK* were studied by PCR and bidirectional direct sequencing. Furthermore, methylation of negative RAS signalling regulator genes, *RASSF1A* and *RASD1*, were analyzed by methylation-specific PCR. As evidenced by phospho-ERK1/2 over-expression, functional RAS activation was detected in 12 (75.0%) RRMM. Of patients with functional RAS activation, sequencing data showed only seven (58.3%) patients with one each had *NRAS* Q61H, *NRAS* Q61K, *KRAS* G12D, *KRAS* G12V, *KRAS* G13D, *KRAS* Q61P, or *BRAF* V600E mutation, whereas five (41.7%) patients had no *RAS*/*RAF* mutation. Conversely, patients without functional RAS activation had no *RAS*/*RAF* mutation. Moreover, none of the patients with functional RAS activation had *ALK* mutations, or methylation of *RASSF1A* and *RASD1*. Collectively, functional activation of RAS signalling was present in majority of RRMM but only about half (58.3%) accountable by *RAS*/*RAF* mutations. If verified in larger studies, clinical investigations of MEK inhibitors are warranted regardless of *RAS*/*RAF* mutations.

## Introduction

RAS signalling pathway plays a key role in the regulation of cellular proliferation^1^. RAS family proteins consist of HRAS, KRAS, and NRAS. In mammalian cells, binding of cytokines, growth factors or mitogens to their cognate surface receptors will lead to activation of the corresponding receptor tyrosine kinases (RTKs) in the intracellular domain, recruitment and hence formation of the cytosolic SHC/GRB2/SOS complex, whereby inactive GDP-bound RAS is converted into active GTP-bound RAS^2^. Activated RAS will sequentially phosphorylate and activate RAF, mitogen-activated protein kinase kinases (MEKs), and extracellular signal–regulated kinases (ERKs)^3^. Activated ERKs may translocate into nucleus, leading to phosphorylation and activation of multiple transcription factors, and hence gene expressions^4^. In cancers, activating mutations of *KRAS* and *NRAS* are frequently found and associated with over-activities of the RAS-RAF-MEK-ERK cascade, resulting in upregulation of pro-survival transcription factors involved in cell cycle progression. Other causes of constitutive activation of RAS signalling, based on phosphorylated ERK1/2 over-expression, may involve activating mutations of *BRAF*^1^ or anaplastic lymphoma kinase (*ALK*) receptor tyrosine kinase (localized to chromosome 2p23)^5^, over-expression of growth factors (such as IL-6)^1^, or methylation-mediated silencing of tumour-suppressive negative regulator genes, such as *RASSF1A*^6^ and *RASD1*^7^, of the RAS signalling pathway.

Multiple myeloma is an incurable haematological malignancy characterized by neoplastic proliferation of clonal plasma cells in the bone marrow^8^. Genetic aberrations, such as t(4;14), t(14;16), deletion 17p13, and amplification of 1q21 [amp(1q21)], are associated with poor prognosis^8^. Activating mutations of RAS signalling pathway have been reported in approximately half of newly diagnosed myeloma and an even higher proportion of relapsed/refractory multiple myeloma (RRMM)^9,10^. However, evidence for functional activation of RAS signalling with over-expression of phospho-ERK1/2 by Western blot in primary myeloma plasma cells is scanty.

Previously, in a bortezomib- and lenalidomide-refractory myeloma patient, we have shown that constitutive activation of RAS signalling, as evidenced by over-expression of phospho-ERK1/2 by Western blot, was attributed by *BRAF* V600E but not *KRAS*/*NRAS* mutation^11^. Herein, we studied the activation of RAS-RAF-MEK-ERK cascade in primary myeloma plasma cells from the CD138-sorted marrow or nodal plasma cells of 17 suspected RRMM by Western blotting of phospho-ERK1/2. Status of RAS signalling activation was then correlated with activating mutations in *KRAS*, *NRAS*, *BRAF*, or *ALK*, amp(1q21), and methylation of *RASSF1A* and *RASD1*.

## Results

**Figure 1:**
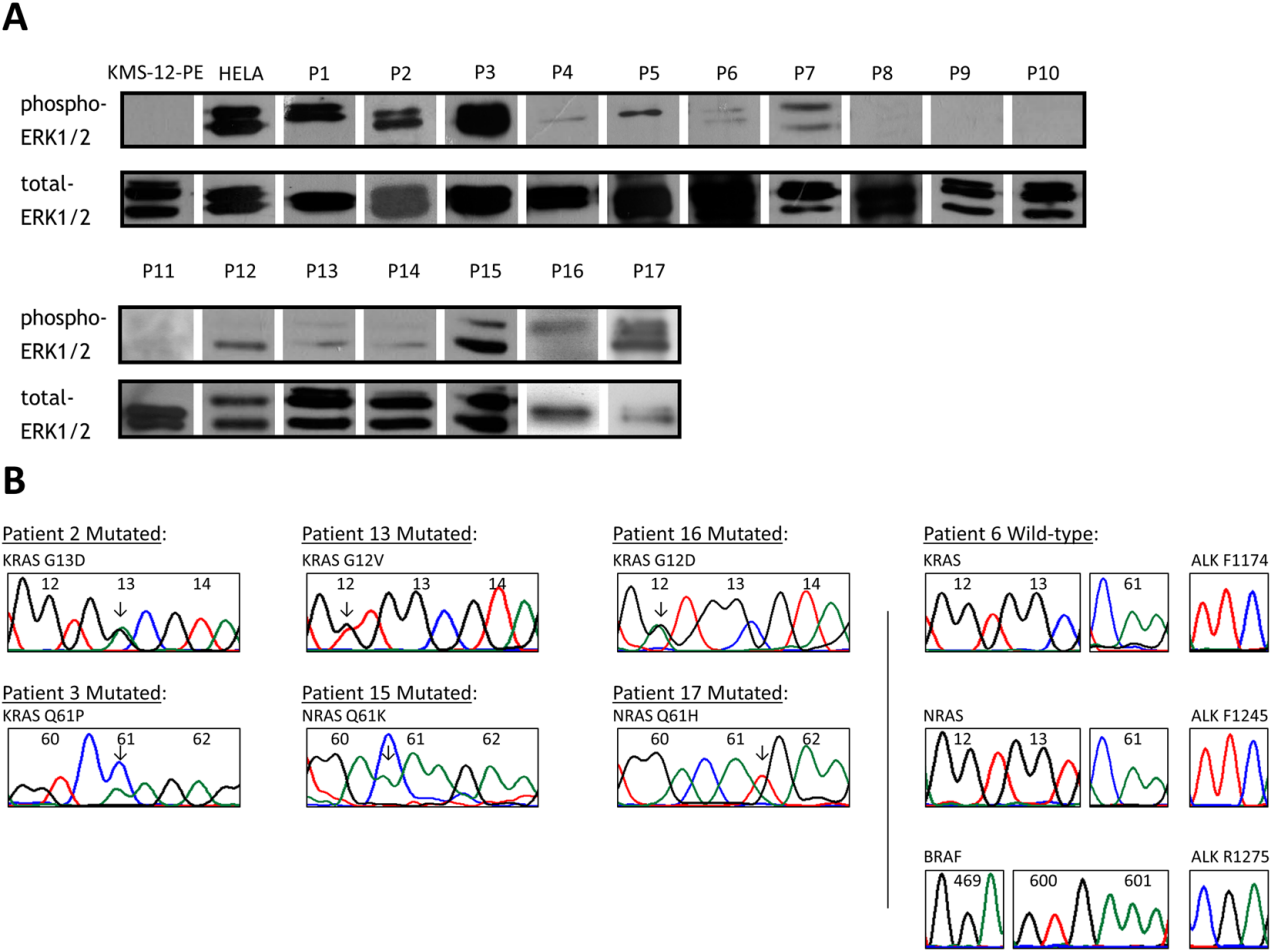
RAS signalling activation in patients with relapsed and/or refractory myeloma. (**A**) Western blot analysis of CD138-sorted bone marrow plasma cells showed ERK1/2 activation. KMS-12-BM served as negative and HeLa cells as positive control for RAS signalling activation based on phospho-ERK1/2 expression. Cropped blots were shown, whereas full-length blots are available upon request. (**B**) Sequencing analysis of *NRAS*, *KRAS*, and *ALK* showed *KRAS* G13D (patient 2), *KRAS* Q61P (patient 3), *KRAS* G12V (patient 13), *NRAS* Q61K (patient 15), *KRAS* G12D (patient 16), and *NRAS* Q61H (patient 17) mutations, whereas wild-type was also illustrated (patient 6).

Of the 17 patients, one patient (P5) had bone marrow examination for suspected myeloma relapse due to onset of pancytopenia, which revealed Philadelphia chromosome-negative acute lymphoblastic leukaemia but no evidence of myeloma, hence was excluded from analysis. By Western blot analysis on primary myeloma plasma cells, 12/16 (75.0%) RRMM patients showed phospho-ERK1/2, indicating constitutive activation of the RAS-RAF-MEK-ERK signalling pathway (Fig. 1A and Table 1). Of the 12 patients with functional ERK activation, seven (58.3%) showed *RAS*/*RAF* mutations with one each had *NRAS* Q61H, *NRAS* Q61K, *KRAS* G12D, *KRAS* G12V, *KRAS* G13D, *KRAS* Q61P, or *BRAF* V600E mutation [this case was reported in Chim *et al*.^11^] (Fig. 1B and Table 1). However, the other five patients with functional ERK activation had no *RAS*/*RAF* mutation at the selected mutation hotspots, including *KRAS*/*NRAS* (codon 12, 13 and 61) and *BRAF* (codon 469 and 600), which account for almost all RAS activation in cancers^1^. Moreover, in the remaining four patients without functional ERK activation, no *RAS*/*RAF* mutation was detected.

**Table 1:** RAS-RAF-MEK-ERK activation, *RAS*/*RAF* mutation, *ALK* mutation, 1q21 amplification, and *RASSF1A*/*RASD1* methylation in patients with RRMM.

Patient	pERK1/2	Amp(1q21)	KRAS codon			NRAS codon			BRAF codon			ALK codon			RASSF1A	RASD1
			12	13	61	12	13	61	469	600	601	1174	1245	1275	Methylation	Methylation
1	**Pos**	**Pos**	WT	WT	WT	WT	WT	WT	WT	WT	WT	WT	WT	WT	UU	UU
2	**Pos**	**Pos**	WT	**G(G/A)C**	WT	WT	WT	WT	WT	WT	WT	WT	WT	WT	UU	UU
3	**Pos**	**Pos**	WT	WT	**C(A/C)A**	WT	WT	WT	WT	WT	WT	WT	WT	WT	UU	UU
4	**Pos**	**Pos**	WT	WT	WT	WT	WT	WT	WT	**G(T/A)G**	WT	WT	WT	WT	UU	UU
5^^^	**Pos**	Neg	WT	WT	WT	WT	WT	WT	WT	WT	WT	WT	WT	WT	UU	UU
6	**Pos**	Neg	WT	WT	WT	WT	WT	WT	WT	WT	WT	WT	WT	WT	UU	UU
7	**Pos**	**Pos**	WT	WT	WT	WT	WT	WT	WT	WT	WT	WT	WT	WT	UU	UU
8	Neg	**Pos**	WT	WT	WT	WT	WT	WT	WT	WT	WT	WT	ND	ND	UU	UU
9	Neg	Neg	WT	WT	WT	WT	WT	WT	WT	WT	WT	WT	WT	WT	UU	UU
10	Neg	**Pos**	WT	WT	WT	WT	WT	WT	WT	WT	WT	WT	WT	WT	UU	UU
11	Neg	N/A	WT	WT	WT	WT	WT	WT	WT	WT	WT	WT	WT	WT	UU	UU
12	**Pos**	N/A	WT	WT	WT	WT	WT	WT	WT	WT	WT	ND	ND	ND	ND	ND
13	**Pos**	N/A	**G(G/T)T**	WT	WT	WT	WT	WT	WT	WT	WT	WT	WT	WT	UU	UU
14	**Pos**	**Pos**	WT	WT	WT	WT	WT	WT	WT	WT	WT	WT	WT	WT	ND	ND
15	**Pos**	**Pos**	WT	WT	WT	WT	WT	**(C/A)AA**	WT	WT	WT	WT	WT	WT	UU	UU
16	**Pos**	N/A	**G(G/A)T**	WT	WT	WT	WT	WT	WT	WT	WT	WT	WT	WT	UU	UU
17	**Pos**	N/A	WT	WT	WT	WT	WT	**CAT**	WT	WT	WT	WT	WT	WT	UU	UU

Keys: RRMM, relapsed/refractory multiple myeloma; Pos, positive; Neg, negative; N/A, not applicable; WT, wild-type; ND, not done due to insufficient DNA; ALK codon 1174/1245/1275 WT, TTC/TTC/CGA; KRAS codon 12/13/61 WT, GGT/GGC/CAA; NRAS codon 12/13/61 WT, GGT/GGT/CAA; BRAF codon 469/600/601 WT, GGA/GTG/AAA; UU, completely unmethylated; ^^^patient in very good partial remission but had bone marrow examination because of pancytopenia, which revealed Philadelphia chromosome-negative acute lymphoblastic leukaemia with no bone marrow plasmacytosis.

Furthermore, alternative mechanism of functional activation of RAS signalling by activating mutations of *ALK* (F1174, F1245, or R1275), which accounted for ERK activation in diagnostic and relapsed neuroblastoma^5^, was not found in our patients with functional activation of RAS (Fig. 1B). In addition, as amp(1q21) is an adverse cytogenetic aberration that may be acquired in myeloma patients at relapse^12^ with overexpression of *CKS1B*^13^, association of amp(1q21) with activation of RAS signalling in myeloma was investigated (Supplementary Fig. S1). However, by FISH in marrow samples at relapse, amp(1q21) was present in seven with and two without phospho-ERK1/2 (*P* = 0.700; Table 1), hence not associated with functional activation of RAS.

**Figure 2:**
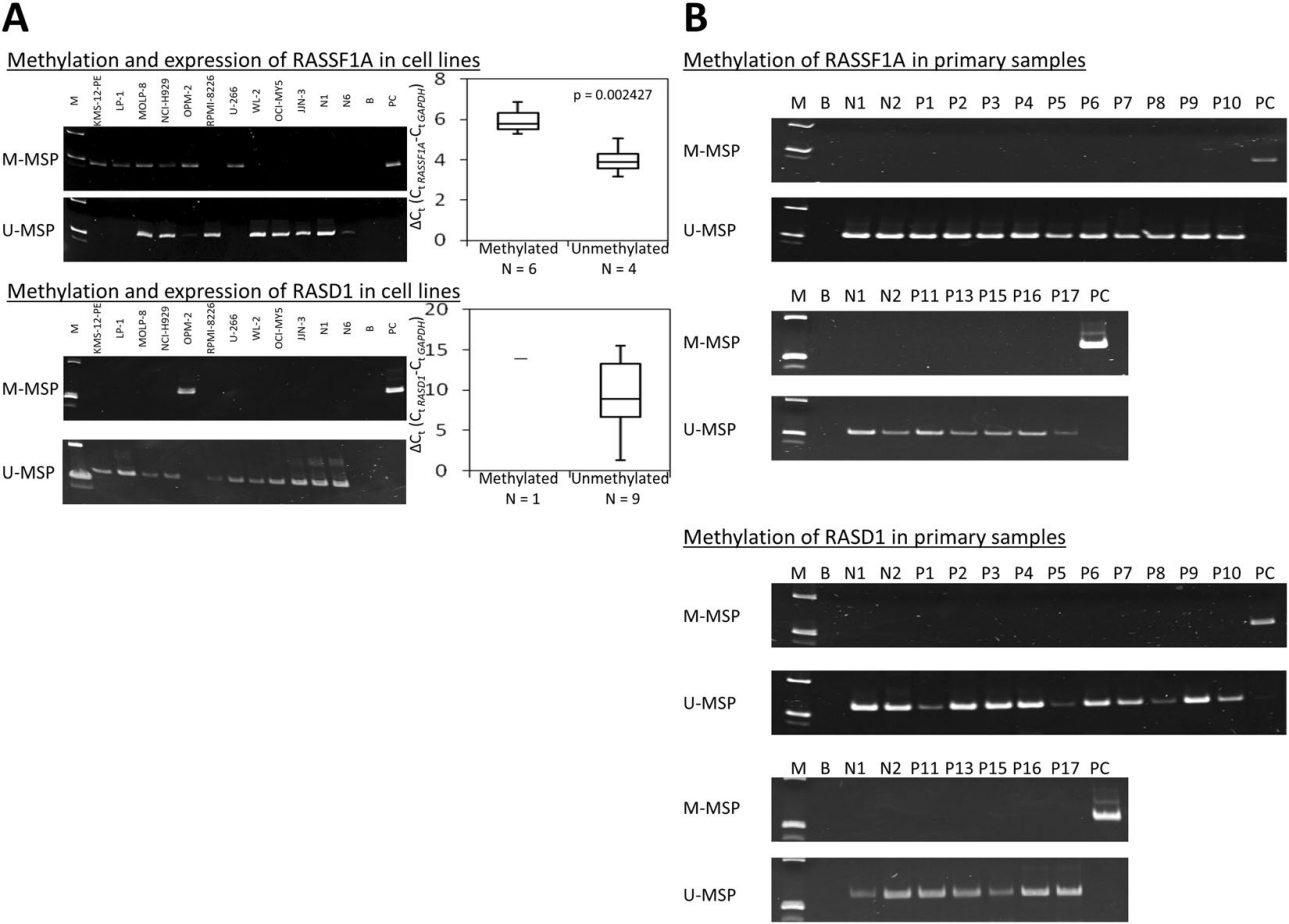
Methylation-mediated silencing of tumour-suppressive negative regulators of the RAS signalling pathway. (**A**) Methylation-specific PCR indicated methylation of *RASSF1A* and *RASD1* in human myeloma cell lines. Quantitative real-time RT-PCR showed an inverse correlation between methylation and expression of each of *RASSF1A* and *RASD1*. (**B**) Methylation-specific PCR showed absence of methylation of *RASSF1A* and *RASD1* in primary samples of patients with relapsed and/or refractory myeloma. M: marker; B: reagent blank; N: normal control; P: patient; PC: positive control with methylated DNA.

Finally, to investigate if methylation-mediated silencing of tumour suppressor genes negatively regulating RAS signalling pathway may account for functional activation of RAS, DNA methylation of *RASSF1A* and *RASD1* promoters were studied by methylation-specific PCR (MSP). Similar to previous reports^6,7^, methylation of *RASSF1A* and *RASD1* were absent in normal controls, including CD138-sorted normal bone marrow plasma cells (n = 8) and normal peripheral blood buffy coats (n = 10) (Supplementary Fig. S2), but detected in six (60%) and one (10%) human myeloma cell lines respectively, hence tumour-specific (Fig. 2A). Moreover, methylation of *RASSF1A* and *RASD1* was associated with low expression in human myeloma cell lines (Fig. 2A), thereby demonstrating methylation-mediated gene silencing. However, in primary samples, neither *RASSF1A* nor *RASD1* methylation was detected (Fig. 2B).

## Discussion

We have demonstrated frequent functional activation of RAS signalling pathway in 75% of RRMM, but only about half (58.3%) accountable by *RAS*/*RAF* mutations. In majority of previous studies, RAS activation, defined and hence inferred by the presence of *RAS*/*RAF* DNA mutations, was present in about 25–55% of newly diagnosed myeloma, and about 45–81% of RRMM^9,10,14–17^. However, our study is the first functional study of RAS activation in primary myeloma samples by Western blot of phospho-ERK1/2.

There is a recent study, in which, functional RAS activation was assessed by immunohistochemistry (IHC) for phospho-ERK1/2, and revealed functional RAS activation in 41.7% RRMM, in which *RAS*/*RAF* mutations were found in 80%^18^. However, the discrepancy in the frequency of functional RAS activation between of both studies might be partly explained by the different definitions of ERK activation between IHC and Western blot. In the IHC study^18^, ERK activation was defined by the presence of median/strong IHC signal for phospho-ERK1/2 (intensity score >1) in ≥30% of tumour cells. On the contrary, in our study, ERK activation was defined by the presence of Western blot signal for phospho-ERK1/2 in protein lysate of CD138-sorted myeloma plasma cells of the patients’ marrow and the positive (HeLa cells) but not negative (KMS-12-PE cells) controls. However, given that the incidence of myeloma is much higher in the Western countries with an incidence of 6.6/100,000/year in the US^19,20^, as compared to about 1.7/100,000/year in the Hong Kong Chinese^21^, a genuine difference in the frequency of RAS activation remains possible. On the other hand, in contrast to the IHC study showing the presence of *NRAS* G13R in cases without functional RAS activation, our data showed absence of *RAS*/*RAF* mutation in all RRMM patients without functional RAS activation, suggesting that functional RAS signaling activation defined by Western blotting of phosphor-ERK1/2 may be more specific. Collectively, our data showed that functional RAS activation was prevalent in RRMM that would otherwise be underestimated by the frequency of *RAS*/*RAF* DNA mutations.

Regarding the mechanism of RAS activation, *RAS*/*RAF* mutation is the most important cause. Indeed, various studies had shown frequent *RAS*/*RAF* mutations in particular in RRMM^9,15^. Other mechanisms of RAS activation include gain-of-function mutation of oncogenes including ALK^22–24^. In contrast to frequent *ALK* mutations accounting for RAS activation in neuroblastoma^5,25^, our study did not demonstrate any *ALK* mutation.

On the other hand, RAS activation may result from loss-of-function of tumour suppressor genes, either by loss of function mutation or promoter DNA methylation of negative regulators of RAS signalling. DNA methylation is an alternative mechanism of gene silencing mediated by addition of methyl groups to the cytosine rings of the CpG dinucleotides in the promoter-associated CpG islands of tumor suppressor genes^26^. In myeloma, multiple tumour suppressor genes and miRNAs have been shown to be associated with methylation-mediated gene silencing^27^. For instance, *RASSF1A* and *RASD1* promoter DNA methylation have been shown in myeloma to be associated with activation of RAS signalling^6,7^. Herein, we showed tumour-specific methylation of both *RASSF1A* and *RASD1* in myeloma cell lines, as evidenced by the presence of methylated MSP signals in myeloma cell lines but not normal control DNA. Moreover, the inverse correlation between *RASD1* methylation and expression was consistent with methylation-mediated gene silencing. Indeed, in a genome-wide methylation study of 115 primary myeloma samples using Illumina 27 K^28^, *RASD1* methylation was inversely correlated with expression, hence further testifying the role of methylation-mediated silencing for *RASD1* in myeloma patients. However, in contrast to the previous reports of *RASSF1A* and *RASD1* hypermethylation in primary myeloma samples^6,7^, *RASSF1A* or *RASD1* methylation was absent in all samples, including those with functional RAS activation. Therefore, methylation of either *RASSF1A* or *RASD1* may not be important for the activation of RAS in RRMM. However, in view of the limited number of samples herein, further studies with larger number of patients are required. Moreover, investigation into additional mechanisms of activation of RAS signalling is warranted. In addition to promoter DNA methylation, RASSF1A inactivation had been shown to be associated with a repressive chromatin configuration with histone deacetylation and H3K9 dimethylation in prostate cancer cell lines^29^.Nonetheless, in primary lung, liver and renal cancer tissues, *RASSF1A* inactivation has been consistently shown to be mediated by promoter DNA methylation, hence an important role in the regulation of *RASSF1A* expression^30^.

Finally, as our study showed functional activation of RAS in the majority (75%) of RRMM regardless of the presence of *RAS*/*RAF* mutation, activated RAS signaling poses a potential therapeutic target in RRMM. Moreover, as functional activation of RAS may occur with either *RAS* or *RAF* mutations, inhibition of RAS signalling appears more effective by the use of inhibitors to downstream signalling effectors such as MEK or ERK1/2. Indeed, there are ongoing clinical trials using MEK inhibitor, trametinib, in RRMM, which appeared promising^31,32^.

## Conclusions

Collectively, functional activation of RAS signalling, as evidenced by ERK activation, was present in the majority of RRMM, but only accountable by known *RAS*/*RAF* mutations in half. In view of prevalent functional RAS activation regardless of *RAS*/*RAF* mutations, a clinical trial of MEK inhibitors is warranted in RRMM.

## Methods

### Patients

Seventeen patients with suspected RRMM, including those with “relapsed” or “relapsed-and-refractory myeloma” were included for functional study of activation of RAS-RAF-MEK-ERK cascade by Western blot of phospho-ERK1/2 in primary myeloma plasma cells from the CD138-sorted marrow or nodal plasma cells.

Relapsed myeloma is defined as previously treated myeloma that progresses and requires the initiation of salvage therapy but does not meet criteria for either “primary refractory myeloma” or “relapsed-and-refractory myeloma” categories^33^. Relapsed and refractory myeloma is defined as disease that is nonresponsive while on salvage therapy, or progresses within 60 days of last therapy in patients who have achieved minimal response (MR) or better at some point previously before then progressing in their disease course^33^.

Patient demographics were described in Supplementary Table S1. This study has been approved by the Institutional Review Board of Queen Mary Hospital with informed consent in accordance with the Declaration of Helsinki. All study methods were performed in accordance with relevant guidelines and regulations.

### Cell cultures

Human myeloma cell lines LP-1 and RPMI-8226 were kindly provided by Prof. Robert Orlowski (Department of Lymphoma/Myeloma, Division of Cancer Medicine, The University of Texas MD Anderson Cancer Center, Houston, TX, USA), JJN-3 and OCI-MY5 by Prof. Wee Joo Chng (Department of Medicine, Yong Loo Lin School of Medicine, National University of Singapore), and WL-2 by Prof. Andrew Zannettino (Myeloma Research Programme, The University of Adelaide, Australia). NCI-H929 was purchased from American Type Culture Collection (Manassas, VA, USA). Other myeloma cell lines (KMS-12-PE, MOLP-8, OPM-2 and U-266) were purchased from Deutsche Sammlung von Mikroorganismen und Zellkulturen (DSMZ) (Braunschweig, Germany). Cells were cultured in RPMI-1640 medium (IMDM for LP-1), supplemented with 10% fetal bovine serum, 50 U/ml of penicillin and 50 ug/ml streptomycin, and incubated in a humidified atmosphere of 5% CO_2_ at 37 °C. All cell culture reagents were purchased from Invitrogen (Carlsbad, CA, USA).

### Western blotting for ERK1/2 activation

Purified CD138+ plasma cells obtained from magnetic-activated cell sorting (Miltenyi Biotec, Cologne, Germany) were lysed in RIPA buffer supplemented with phosphatase inhibitor cocktail and 1 mM PMSF. Cell debris was removed by centrifugation at 10,000 × g for 5 min at 4 °C. Protein lysate was heated in an equal volume of blue loading buffer at 95 °C for 5 min, and separated on 10% SDS-PAGE. Separated samples were then transferred to a 0.45 μm PVDF membrane (Amersham Biosciences, Buckinghamshire, UK). The membrane was blocked at room temperature for 1 hour in 5% skim milk diluted in PBS-Tween 20 (0.5% v/v). The membrane was then incubated with specific primary antibody (1:1000) (Cell signalling, Danvers, MA, USA) at 4 °C overnight with shaking. After washing 3 times of 15 minutes each in PBS-Tween 20 (0.5% v/v), the membrane was incubated with specific horseradish peroxidase conjugate secondary antibody (1:1000) (Bio-Rad) at room temperature for 1 hour. After washing 3 times of 15 minutes each in PBS-Tween 20 (0.5% v/v), signals were detected by ECL Western blotting detection reagents (Amersham Biosciences, Buckinghamshire, UK) and exposed to X-ray film.

### Mutation analysis of *KRAS*, *NRAS*, *BRAF*, and *ALK*

Direct sequencing analysis was employed to study codons 12, 13, and 61 of *KRAS* and *NRAS*, which encompass more than 96% of all *RAS* mutations in human cancers^34^; to examine codons 469, 600 and 601 of *BRAF*, which represent more than 85% of all *BRAF* mutations in human cancers^35^; and to study codons 1174, 1245, 1275 of *ALK*, which account for 85% of *ALK* mutations in diagnostic neuroblastoma^36^. In brief, genomic DNA extracted from CD138-sorted plasma cells was amplified by PCR, followed by bidirectional direct sequencing. Primer sets specific to these regions adopted from literatures were shown in Supplementary Table S2 ^5,35,37^. Results were compared to reference sequences NM_004304 (*ALK*), NM_004985 (*KRAS*), NM_002524 (*NRAS*) and NM_004333 (*BRAF*).

### Fluorescence *in situ* hybridization (FISH)

Detection of cytogenetic abnormalities was performed on myeloma cells in the bone marrow by FISH, as previously described^38^. Interphase FISH was performed on slides for the examination of high-risk (HR) karyotypes, including t(4;14), t(14;16), del(17p), and amp(1q21), by IGH/FGFR3 DF FISH Probe kit (Vysis, USA), IGH/MAF DF FISH Probe kit (Vysis, USA), TP53/CEP17 FISH Probe kit (Vysis, USA), and CKS1B/CDKN2C (P18) amplification/deletion probe (Cytocell) respectively, in accordance with the International Myeloma Workshop Consensus recommendation. At least 200 nuclei were analyzed and scored independently by two persons. The cutoff for positivity was above 5% or at least 10 positive nuclei based on test validation data.

### Methylation-specific PCR (MSP) for *RASSF1A* and *RASD1*

Genomic DNA of CD138-sorted marrow plasma cells was extracted by QIAamp DNA Blood Mini Kit (Qiagen, Germany), followed by bisulfite conversion of unmethylated cytosine to uracil (but unaffecting methylated cytosine) using EpiTect Bisulfite Kit (Qiagen, Germany). Healthy bone marrow donor DNA and enzymatically methylated control DNA (CpGenome Universal Methylated DNA, Chemicon) served as negative control and positive control respectively in all PCR. Primers used for the methylated MSP (M-MSP) and unmethylated MSP (U-MSP) are shown in Supplementary Table S2. MSP was performed in a Veriti thermal cycler (Applied Biosystems, Foster City, CA). The MSP mixture contained 50 ng of bisulfite-treated DNA, 0.2 mM dNTPs, MgCl2, 10 pmol of each primer, 1 X PCR buffer, and 2.5 units of AmpliTaq Gold DNA Polymerase (ABI, Foster City, CA) in a final volume of 25 μl. Five microliters of PCR products were loaded onto 6% non-denaturing polyacrylamide gels, electrophoresed, and visualized under ultraviolet light after staining with ethidium bromide.

### Quantitative real-time reverse transcription-PCR (qRT-PCR)

In myeloma cell lines, expression of *RASSF1A* or *RASD1* was studied by SYBR Green-based qRT-PCR. In brief, total RNA was isolated using mirVana™ miRNA Isolation Kit (Ambion, Austin, TX, USA), followed by reverse transcription to cDNA using QuantiTect Reverse Transcription Kit (Qiagen), according to the manufacturers’ instructions. The resulting cDNA was used as template for qRT-PCR (iQ SYBR Green Supermix, Bio-Rad), with *GAPDH* as endogenous control. Primer sequences were listed in Supplementary Table S2. Correlation between methylation and expression was studied by Student’s t-test (two-tailed), whereas P < 0.05 was regarded as statistical significant.

All data generated or analysed during this study are included in this published article (and its Supplementary Information files).

### Ethics approval and informed consent

The study has been approved by Institutional Review Board of Queen Mary Hosital (UW 05-269T/932), and written informed consent was obtained from patient for publication of this study and any accompanying data or images.

## Electronic supplementary material

Supplementary Tables and Figures
