# Supplementary material for: Frequent functional activation of RAS signalling not explained by RAS/RAF mutations in relapsed/refractory multiple myeloma
Source: Sci Rep. 2018 Sep 10;8:13522. doi: 10.1038/s41598-018-31820-9 (PMC6131153; doi:10.1038/s41598-018-31820-9)
Supplement: Supplementary file 1 — Supplementary Tables and Figures [file 41598_2018_31820_MOESM1_ESM.pdf]

Supplementary Information

Frequent functional activation of RAS signalling not explained by RAS/RAF mutations in relapsed/refractory multiple myeloma

Kwan Yeung WONG, Qiumei YAO, Ling-Qing YUAN, Zhenhai LI, Edmond Shiu Kwan MA, Chor Sang CHIM*

*Corresponding author: Prof. Chor Sang CHIM, MD, PhD, Department of Medicine, Queen Mary Hospital, The University of Hong Kong, Pokfulam Road, Pokfulam, Hong Kong.

Email: jcschim@hku.hk

Table S1 Patient demographics

Patient	Gender	Isotype	Cytogenetic abnormality	BM status	BMPC%	Regimen prior BM aspiration	Regimen post BM aspiration	Survival since BM study (Month)#
1	M	L	del(17p); amp(1q21)	Refractory	>90%	VTD; auto-BMT; VRD; V-mel-cyclophoshamide-dex	Velcade+Mini-BEAM; Velcade-Dex-Sorfafenib; V-Benda-dex	5
2	M	G	amp(1q21)	Refractory	>80%	VTD; VRD; VCD; auto-BMT	Velcade+mini-BEAM	1
3	F	L	amp(1q21)	Refractory	87%	VAD; auto-BMT; VTD	VCD; VRD; VTD	9
4	M	G	amp(1q21)	Refractory	100%	VTD; VRD; auto-BMT; vel-ATO; vel-sorafenib	VAD; v-Benda-dex; v-POM-dex; RIC allo-BMT	20
5^	M	L	del(17p)	Myeloma in CR → ALL	80% ALL blast	N/A	N/A	N/A
6	F	G	del(17p)	Relapse	7%	VTD; auto-BMT; velcade	VCD; RD; POM-Dex	42
7	M	G	amp(1q21)	Relapse	34%	MP; MPT; Vel-Panobinostat-Dex	Ixazomib(MLN 9708)-RD	39
8	M	A	t(4;14); del(17p) amp(1q21)	Refractory	>80%	MPT; VTD; VCD; Vel-thal-dex-Mel	V-benda-Dex	13

9	F	L	Nil	Relapse	60%	Doxorubicin-Dexa; VTD; Auto-BMT; RD; V-ATO/ascorbic acid	POM-Dex; Melphalen-dex; V-benda-Dex; CTX-DEX	42
10	F	G	del(17p); amp(1q21)	Relapse	35%	VAD; auto-BMT	RD; VCMD	16
11	M	L	Nil	Relapse	N/A	VTD; auto-PBSCT; Len; arsenic, VSD; VPD; R-DHAP	R-POM; velcade	11
12	F	G	Nil	Relapse	N/A	Thal-Dex; auto-BMT; Thal	Velcade; VTD; Len-Dex; POM-Dex; 2nd ASCT;	49
13	F	G	Nil	Relapse	38%	VBCMP; Thal-dex	MLN 9708/dex; auto-PBSCT; Rd	48
14	M	G	t(4;14); amp(1q21)	Refractory	26%	Thal; Pred; Zometa; MPT	Velcade, Len	6
15	M	G	amp(1q21)	Relapse	51%	VAD; auto-BMT; Thal; Arsenic, vel-thal-dex; Len-Dex; Vel-Len-Dex; Dex	Velcade-CMP	7
16	M	G	Nil	Refractory	>80%	VCD; VRD; KPD; daratumumab; Len	Dara-Ixa-Benda-Dex	2
17	M	G	Nil	Refractory	70%	VTD; Pamidronate; VCD; Ixa-Rd	Dara-Benda-Dex; Vel-POM-Dex	1

Keys: ^, patient in very good partial remission but had bone marrow examination because of pancytopenia, which revealed Philadelphia chromosome-negative acute lymphoblastic leukaemia with no bone marrow plasmacytosis; BMPC, bone marrow plasma cell; CR, complete remission; ALL, acute lymphoblastic leukaemia; N/A, not applicable; #, as of Jan 2018

Table S2 Primer sequences

Gene	Forward (5' to 3')	Reverse (5' to 3')	Reference
Mutation analysis			
ALK codon 1174	AAG ATT TGC CCA GAC TCA GC	TGT CCT TGG CAC AAC AAC TG	[1]
ALK codon 1245	AGA TTT CCC TCC TCT CAC TG	ATG TGA GCC CTT GAG ATC TG	
ALK codon 1275	TAG TGA TGG CCG TTG TAC AC	CCA GGA GAT GAT GTA AGG GA	
KRAS codons 12 & 13	CCT GCT GAA AAT GAC TGA AT	TGT TGG ATC ATA TTC GTC CA	[2]
KRAS codon 61	GTA ATT GAT GGA GAA ACC TG	ATA CAC AAA GAA AGC CCT CC	
NRAS codons 12 & 13	CTG GTG TGA AAT GAC TGA GT	GGT GGG ATC ATA TTC ATC TA	
NRAS codon 61	AAG TGG TTA TAG ATG GTG AAA CCT	CAA ATA CAC AGA GGA AGC CTT C	
BRAF codon 469	TCC CTC TCA GGC ATA AGG TAA	CGA ACA GTG AAT ATT TCC TTT GAT	[3]
BRAF codons 600 & 601	TCA TAA TGC TTG CTC TGA TAG GA	GGC CAA AAA TTT AAT CAG TGG A	
Methylation-specific PCR			
RASSF1A M-MSP	GTG TTA ACG CGT TGC GTA TC	AAC CCC GCG AAC TAA AAA CGA	[4]
RASSF1A U-MSP	TTT GGT TGG AGT GTG TTA ATG TG	CAA ACC CCA CAA ACT AAA AAC AA	[4]
RASD1 M-MSP	GAG TCG TAG AGG GAT AGG GGT ATC	CCA AAA TCG AAC TAA AAC TAA AAA CG	
RASD1 U-MSP	GAG TTG TAG AGG GAT AGG GGT ATT	CCA AAA TCA AAC TAA AAC TAA AAA CA	
RT-PCR			
RASSF1A	CAA TGC CCA GAT CAA CAG CA	GGG CAG GTA AAA GGA AGT GC	
RASD1	CCA CCG CAA GTT CTA CTC CAT	CCA GGA TGA AAA CGT CTC CTG T	
GAPDH	GAA GGT GAA GGT CGG AGT C	GAA GAT GGT GAT GGG ATT TC	

References:

1 Chen, Y. *et al.* Oncogenic mutations of ALK kinase in neuroblastoma. *Nature* **455**, 971 (2008).

2 Neri, A., Knowles, D. M., Greco, A., McCormick, F. & Dalla-Favera, R. Analysis of RAS oncogene mutations in human lymphoid malignancies. *Proceedings of the National Academy of Sciences* **85**, 9268-9272 (1988).

3 Davies, H. *et al.* Mutations of the BRAF gene in human cancer. *Nature* **417**, 949-954 (2002).

4 Ng, M. H. *et al.* Alterations of RAS signalling in Chinese multiple myeloma patients: absent BRAF and rare RAS mutations, but frequent inactivation of RASSF1A by transcriptional silencing or expression of a non-functional variant transcript. *British Journal of Haematology* **123**, 637-645 (2003).

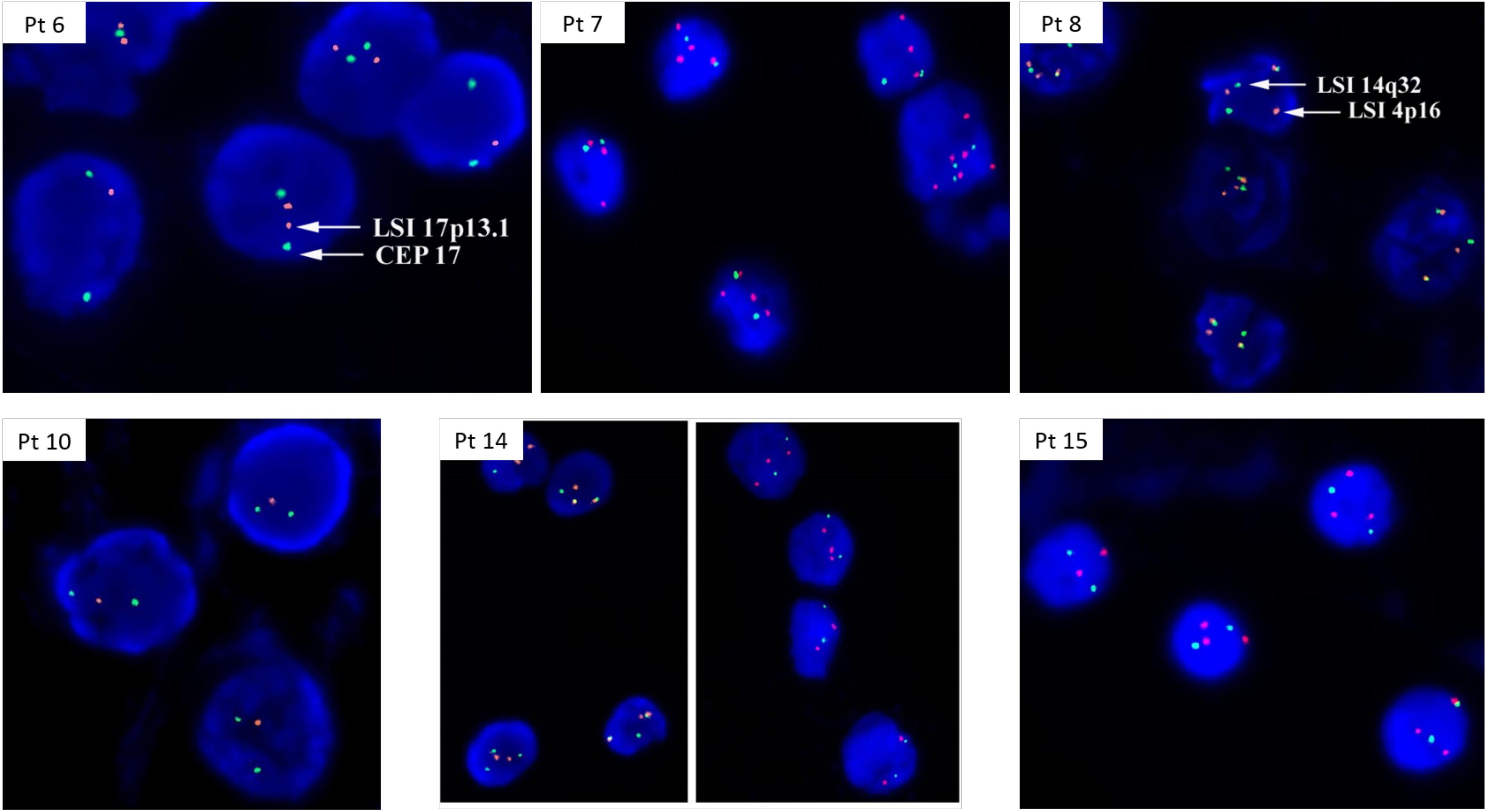

Supplementary figure S1. Images of FISH probe hybridized plasma cells. Pt 6 and Pt 10: TP53/CEP17 probe, showing 1R2G signal pattern on the plasma cells positive for del(17p)/TP53; Pt 7, Pt 14 (right) and Pt 15: CKS1B/CDKN2C(P18) probe, showing 3R2G signal pattern on the plasma cells positive for +1q21; Pt 8 and Pt 14 (left): t(4;14) IGH/FGFR3 probe, showing the 2F1R1G signal pattern positive plasma cells.

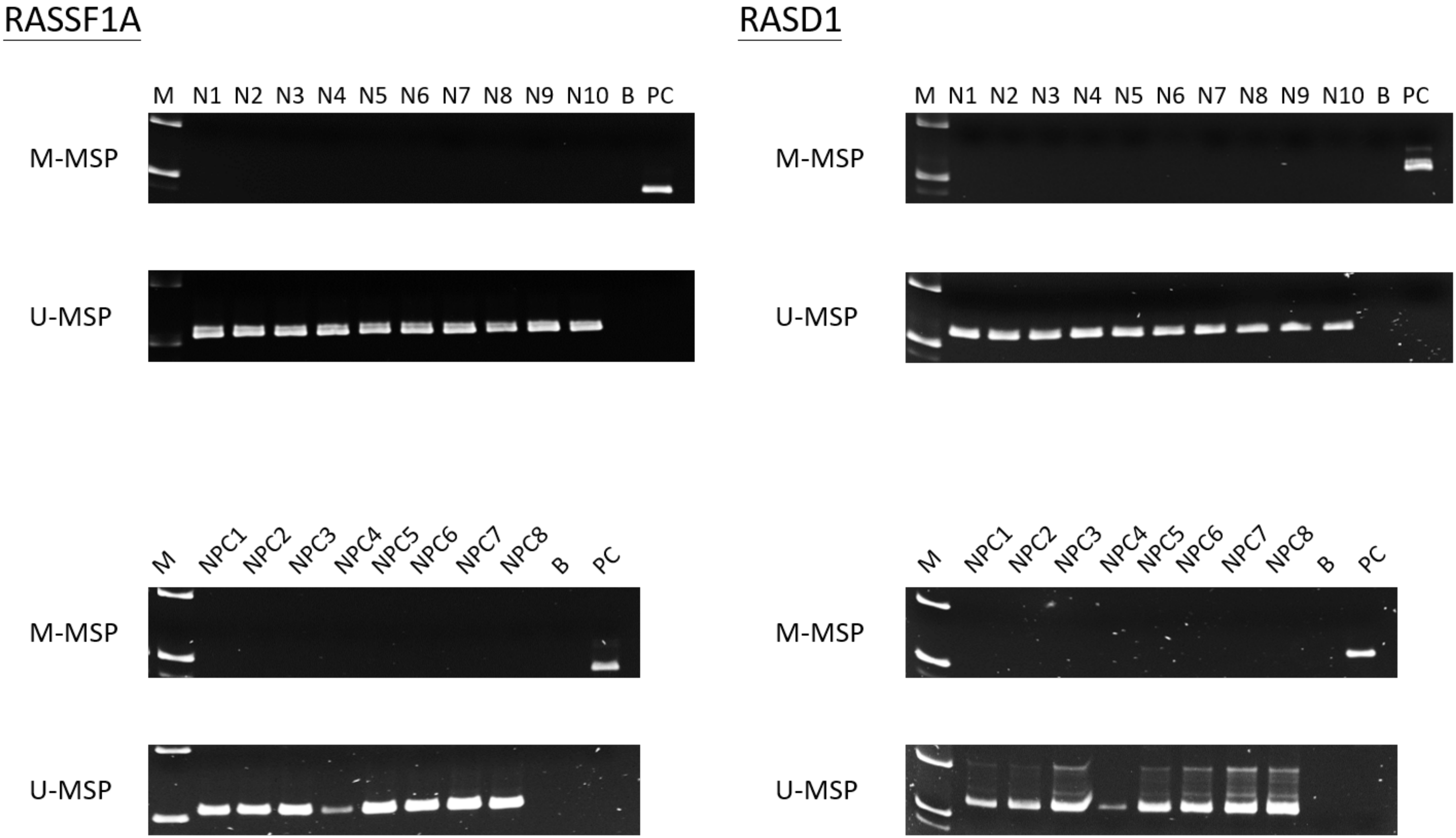

M: marker; N1-N10: normal peripheral blood buffy coat controls; NPC1-NPC8: normal CD138-sorted bone marrow plasma cells; B: no template control; PC: positive control with methylated DNA

Supplementary figure S2. Methylation of RASSF1A and RASD1 in normal controls.
